# Severe bicompartmental bone bruise is associated with rotatory instability in anterior cruciate ligament injury

**DOI:** 10.1007/s00167-021-06735-0

**Published:** 2021-09-07

**Authors:** Piero Agostinone, Stefano Di Paolo, Gian Andrea Lucidi, Giacomo Dal Fabbro, Alberto Grassi, Stefano Zaffagnini

**Affiliations:** 1 Clinica Ortopedica e Traumatologica II, IRCCS Istituto Ortopedico Rizzoli, Via Pupilli 1, 40136 Bologna, BO Italy; 2 Dipartimento di Scienze per la Qualità della Vita QUVI, Università Di Bologna, Corso D’Augusto 237, 47921 Rimini, RN Italy

**Keywords:** Bone bruise, Pivot shift, Surgical navigation, ACL injury

## Abstract

**Purpose:**

The presence and severity of bone bruise is more and more investigated in the non-contact anterior cruciate ligament (ACL) injury context. Recent studies have advocated a correlation between bone bruise and preoperative knee laxity. The aim of the present study was to investigate the correlation between bone bruise and preoperative rotatory knee laxity.

**Methods:**

Twenty-nine patients (29.1 ± 9.8 years) with MRI images at a maximum of 3 months after ACL injury (1.6 ± 0.8 months) were included. The bone bruise severity was evaluated according to the International Cartilage Repair Society (ICRS) scale for lateral femoral condyle, lateral tibial plateau, medial femoral condyle, and medial tibial plateau. The intraoperative rotational knee laxity was evaluated through a surgical navigation system in terms of internal–external rotation at 30° and 90° of knee flexion (IE30, IE90) and internal–external rotation and acceleration during pivot-shift test (PS IE, PS ACC). The KOOS score was also collected. The association between ICRS grade of bone bruise and rotational laxity or KOOS was investigated.

**Results:**

Significant correlation (*p* < 0.05) was found between the bone bruise severity on the medial tibial plateau and rotational laxity (IE90, PS IE, and PS ACC) and between the severity of bone bruise on femoral lateral condyle and KOOS-Symptoms sub-score. The presence of bone bruise on the medial tibial plateau was significantly associated with a lateral femoral notch sign > 2 mm (very strong odds ratio). No kinematical differences were found between none-to-deep and extensive-generalized lateral bone bruise, while higher IE30 and IE90 were found in extensive-generalized bicompartmental bone bruise than isolated extensive-generalized lateral bone bruise.

**Conclusion:**

A severe bicompartmental bone bruise was related to higher rotatory instability in the intraoperative evaluation of ACL deficient knees. The severity of edema on the medial tibial plateau was directly correlated with higher intraoperative pivot shift, and the size of edema on the lateral femoral condyle was associated with lower preoperative clinical scores.

**Level of evidence:**

Level II.

**Supplementary Information:**

The online version contains supplementary material available at 10.1007/s00167-021-06735-0.

## Introduction

Residual rotational instability after anterior cruciate ligament (ACL) reconstruction has been associated with lower clinical scores, impaired sport participation, and increased revision rate [1, 11, 23]. The current target of the orthopedic surgeons is to identify factors that can predict higher rotatory knee laxity after ACL tear to select patients that could benefit from additional procedures [10, 23].

The assessment of rotatory knee laxity through radiological signs might represent an agile and user-friendly approach for preoperative screening. Bone bruise (BB) represents trabecular bone marrow edema that could be identified in up to 80% of acute ACL tear MRI, mainly in non-contact injury mechanisms [9, 12, 24, 28, 32]. The prevalence and location of BB in the context of ACL injury were investigated [17, 28] as well as the association with meniscal lesions [2, 19] and chondral lesions [9, 20]. Moreover, BB’s prognostic role in long-term clinical outcomes has been speculated [9].

Recently, a relationship between lateral bone edema and preoperative rotatory knee laxity was advocated. Song et al. [30] reported a correlation between moderate–severe BB of the lateral femur and tibia and high-grade pivot-shift, lateral meniscus tear, and anterolateral ligament (ALL) abnormality. Under similar hypothesis and methodology, Marot et al. [24] excluded a relationship between bone contusions and ALL tears or rotatory knee instability in a more recent study. The latter studies’ main limitation was that knee laxity evaluation was based on clinical examination and not on instrumented quantitative measurements. Furthermore, only the lateral compartment was investigated, thus overlooking a possible role of the medial bruises on knee laxity.

Thus, the present study aimed to investigate the relation between BB and intraoperative knee laxity quantified with the surgical navigation system. The hypothesis were: (I) lateral BB is associated with higher rotatory instability; (II) bicompartmental (medial plus lateral) BB further increases rotatory instability.

## Materials and methods

### Ethics

This study obtained approval from the Institutional Review Board (IRB) of Rizzoli Orthopaedic Institute (ID: 40/CE/US/ml—Clinical Trial Gov ID: NCT02323386. All subjects signed informed consent before participating in the study.

### Patient selection

This study represents the secondary analysis of data collected from a prospective study aimed to evaluate the outcome of ACL reconstruction. Based on the study protocol of this prospective study, 97 patients were included and assessed preoperatively with a 1.5 T MRI.

The inclusion criteria for the original study were: age 16–50 years, complete, traumatic, and unilateral ACL injury, no previous knee ligament reconstruction or repair, no concomitant lesions of other ligaments, and absence of mild or advanced knee osteoarthritis (Kellgren–Lawrence III–IV).

For the purpose of the present study, only the patients fulfilling the following criteria were selected and further analyzed: non-contact ACL injury; time from injury to MRI < 3 months, complete intraoperative kinematical assessment with surgical navigation system.

Of the 97 patients included in the original study, 29 matched the inclusion criteria and were included in the study. The patients’ mean age was 29.1 ± 9.8 years (range 16–47), and the time from injury to MRI was 1.6 ± 0.8 months (range 0.2–2.9). The complete demographics are reported in Table 1.

**Table 1:** Patients’ demographics

Gender	
Male	24 (83%)
Female	5 (17%)
Injured leg	
Right	18 (62%)
Left	11 (38%)
Body mass index	
< 24	10 (35%)
> 24	19 (65%)
Medial meniscus lesion	
Yes	12 (41%)
No	17 (59%)
Lateral meniscus lesion	
Yes	8 (28%)
No	21 (72%)
Lateral femoral notch sign > 2 mm	
Yes	4 (14%)
No	25 (86%)

### Bone bruise severity according to the IRCS grading

**Fig. 1:**
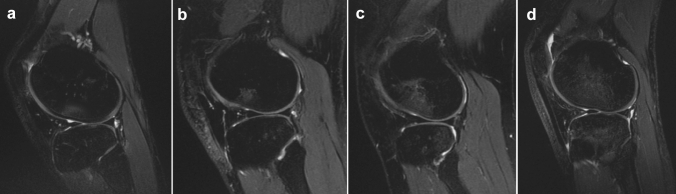
Bone bruise grading system according to ICRS (International Cartilage Repair Society): **A** shallow—grade 1, extending as far as one-third of the distance from the articular surface to the physeal scar; **B** deep—grade 2, extending between one-third and two-thirds of the distance to the physeal scar; **C** extensive—grade 3, extending at least two-thirds of the distance to the physeal scar but not beyond the scar; **D** generalized—grade 4, extending beyond the physeal scar

The presence and severity of bone contusion were determined through preoperative 1.5 T MRI. Sagittal images were reviewed by two different investigators (X.X., X.X.), blinded to the purpose of the study. Proton density fat-suppressed sequence was used to identify better and quantify the bone marrow edema. Each anatomical side of the knee (lateral tibial plateau, lateral femoral condyle, medial tibial plateau, and medial femoral condyle) was separately analyzed by the two investigators, who independently registered BB presence and conducted the International Cartilage Repair Society (ICRS) grading, as described by Brittberg and Winalski [3] (Table 2). The grading was performed in the sagittal slice, in which the largest amount of bone contusion is present for each anatomical side. The detailed grading system is reported in Fig. 1.

**Table 2:** Bone bruise incidence and severity (*n* = 29)

	Lateral femoral condyle	Medial femoral condyle	Lateral tibial plateau	Medial tibial plateau
Present/absent, *n* (%)	21 (72%)	5 (17%)	24 (83%)	16 (55%)
Severity of bone contusion, *n* (%)				
Grade 0, none	8 (28%)	24 (83%)	5 (17%)	13 (45%)
Grade 1, shallow	4 (14%)	0 (0%)	1 (3%)	4 (14%)
Grade 2, deep	8 (28%)	3 (10%)	5 (17%)	6 (21%)
Grade 3, extensive	5 (17%)	2 (7%)	11 (38%)	4 (14%)
Grade 4, generalized	4 (14%)	0 (0%)	7 (24%)	2 (7%)

Severity of bone bruise according to the IRCS scale. Data are presented as a percentage over the total

### Intraoperative kinematical assessment and patient-reported outcomes

**Fig. 2:**
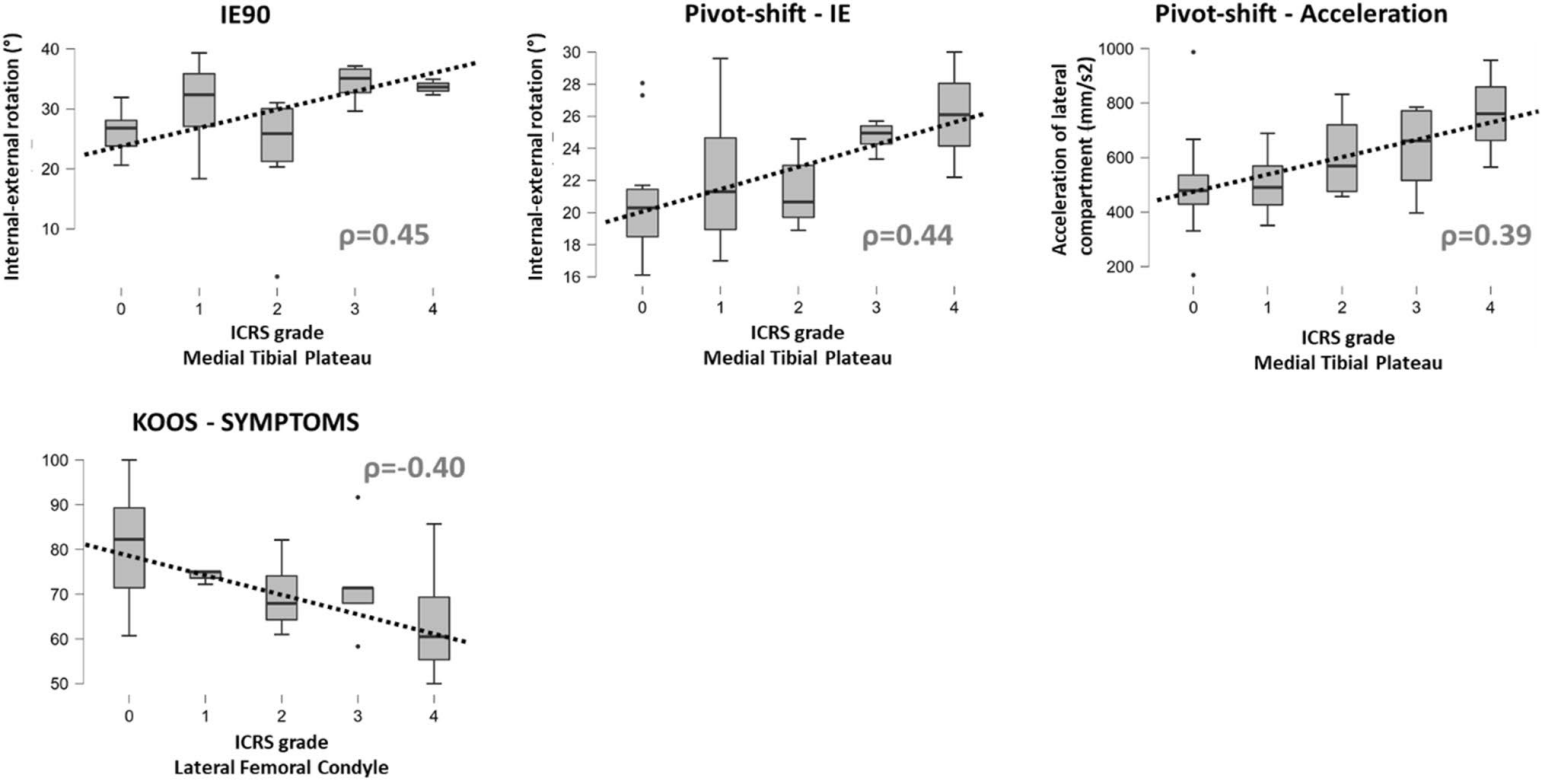
Significant rank correlations (*ρ*) between the bone bruise ICRS grade and rotatory laxity or clinical outcomes: note the direct positive correlation between the severity of medial tibial plateu BB and the intraoperative rotatory laxity and the negative correlation between severity of lateral femoral condyle BB and the preoperative KOOS-Symptoms

A surgical navigation system (BLU-IGS, Orthokey, Lewes, Delaware, DE, USA), equipped with software specifically dedicated to intraoperative kinematics acquisitions (KLEE, Orthokey, Lewes, Delaware, DE, USA) (Fig. 2), was used. The examination protocol was performed in the ACL-deficient status according to the method developed by Martelli et al. [25, 26]. A single experienced orthopedic surgeon (X.X.) manually performed the following clinical kinematic tests at maximum force: internal/external rotation at 30° of flexion (*IE30*); internal/external rotation at 90° of flexion (*IE90*); Pivot-shift (PS) test. According to literature [22], the pivot-shift test was quantified through two parameters: the internal/external rotation of the lateral tibial compartment (named *PS_IE*) and the posterior acceleration of the lateral tibial compartment during tibial reduction (named *PS_ACC*).

The reliability of all laxity tests performed at maximum force was evaluated by a research group in previous studies [21, 25, 26]. Since the present analysis was a secondary investigation of pre-acquired data, the examiner was blind to test quantitative results. Data were elaborated offline with a specifically developed MATLAB interface (The MathWorks Inc, Natick, Massachusetts, USA).

The Knee Injury and Osteoarthritis Outcome Score (KOOS) was administrated before surgery. The sub-scores PAIN, SYMPTOMS, ADL (Activity of Daily Living), SPORT, QoL (Quality of Life) were computed per each patient [5].

### Statistical analysis

The Shapiro–Wilk test was used to inspect the normal distribution of the data. The continuous variables were presented as mean and standard deviations (range), while the categorical variables were presented as a percentage over the total.

The intraclass correlation coefficient (ICC) was used to inspect inter-rater reliability for the assessment of the IRCS grading. Reliability was considered poor, moderate, good, and excellent for ICC values lower than 0.50, between 0.50 and 0.75, between 0.75 and 0.90, and greater than 0.90, respectively [18].

The Spearman’s coefficient *ρ* was used to assess the rank correlation between the presence of BB on the four anatomical sides and the kinematical parameters and clinical sub-scores.

Multivariable logistic regression was also performed to inspect the association between BB and demographical/radiographical findings: the presence/absence of BB in the four anatomical sides were used as end points and gender, injured leg, BMI (< 24 vs > 24), presence of lateral femoral notch sign > 2 mm (threshold for increased rotatory knee laxity [23]), presence of medial meniscus lesion, and presence of lateral meniscus lesion as independent variables. Adjusted odds ratios were estimated for each independent variable. For conciseness, only the statistically significant associations between the anatomical sides and independent variables were reported.

Furthermore, patients were grouped according to the laterality and the severity of the bone bruise in the medial/lateral none-to-deep (grade 0–1-2) and extensive-generalized (grade 3–4) group. To inspect Hypothesis 1, lateral (femoral, tibial, or both) none-to-deep and lateral extensive-generalized groups were compared. To inspect Hypothesis 2, the extensive-generalized isolated lateral bone bruise and extensive-generalized bicomparmental bone bruise groups were compared. The Mann–Whitney *U* test was used to assess the statistical differences between the groups per each of the kinematical parameters.

Differences were considered statistically significant for *p* < 0.05. For the Mann–Whitney *U* test, the rank-biserial correlation coefficient was reported as a measure of effect size. A post hoc power analysis was performed in G*Power (v3.1, Brunsbüttel, Germany) to ensure the statistical effectiveness of the differences obtained for both the correlation analysis (population: *n* = 29) and the group comparisons (minimum number of subjects in a group: *n* = 7). A minimum post hoc power of 72% with *α* = 5% was found for the lowest statistically significant correlation coefficient (PS_ACC, *ρ* = 0.39). Statistical analysis was conducted in JASP (v0.14.1, University of Amsterdam).

## Results

The inter-rater reliability of the ICRS grading was good to excellent for all the four anatomical sides (ICC range 0.87–0.95).

A significant positive correlation (*p* < 0.05) was found between the BB severity on the medial tibial plateau (Fig. 2). A significant negative correlation was found between the severity of BB on the femoral lateral condyle and KOOS-Symptoms sub-score (Fig. 2). Detailed rank correlations can be found in Appendix A.

**Fig. 3:**
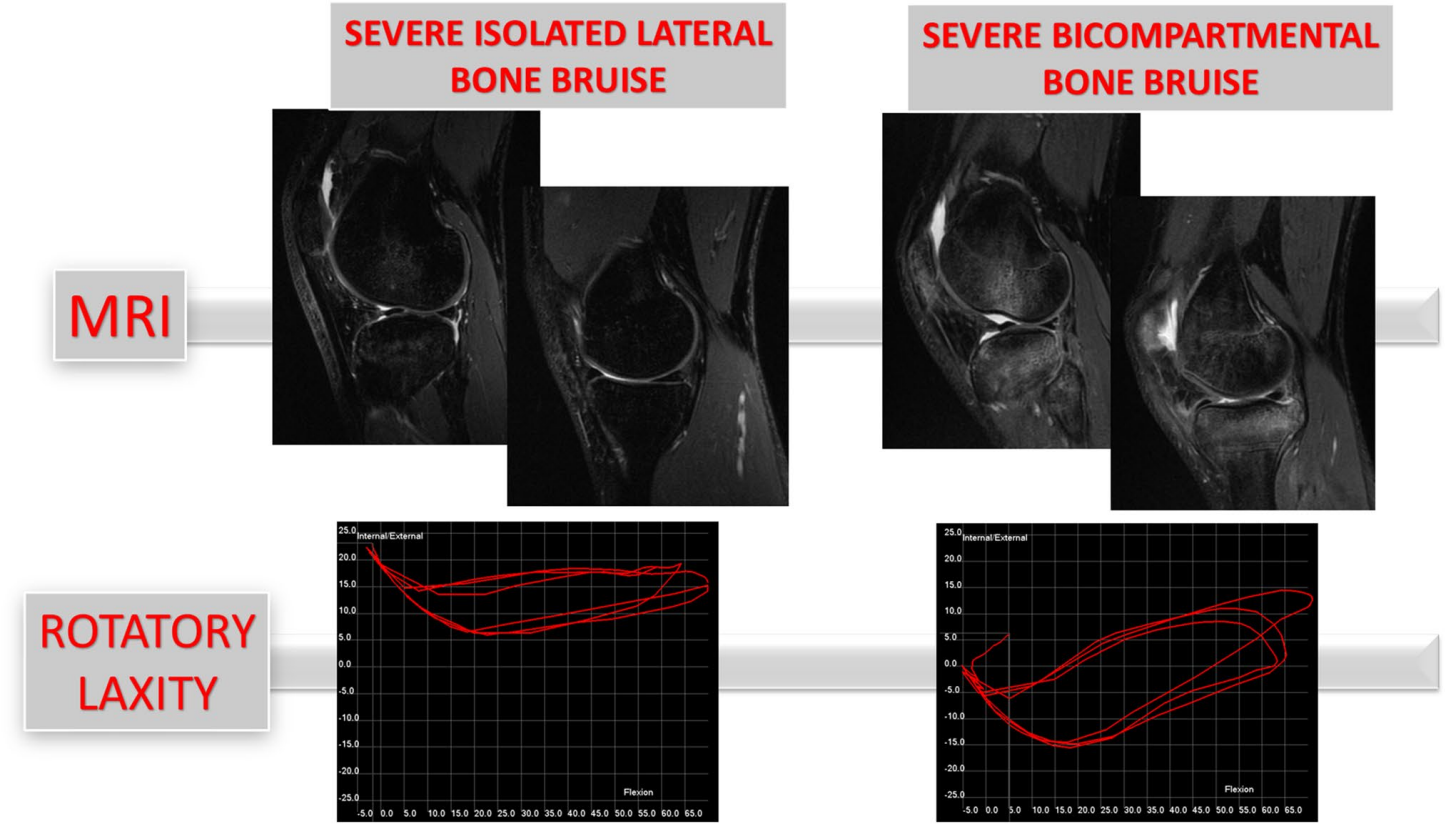
Comparison of rotatory parameters between severe isolated lateral BB and severe bicompartmental BB: the presence of severe bicompartmental BB increases the rotatory laxity assessed by the surgical navigation system

The presence of BB on the medial tibial plateau was associated with the presence of lateral femoral notch sign > 2 mm (Odds Ratio 31.6). Meniscal lesions were not associated with BB of the correspondent compartment. No differences (n.s.) were found between the none-to-deep and extensive-generalized lateral BB for the kinematical parameters (Table 3). Isolated extensive-generalized lateral BB and extensive-generalized bicompartmental BB differed for IE30 and IE90 (Table 4, Fig. 3).

**Table 3:** Intraoperative kinematics according to the severity of lateral bone bruise

	None-to-deep lateral bone bruise (*n* = 11)	Extensive-generalized lateral bone bruise (*n* = 16)	Rank-biserial correlation	*p* value
IE 30 (°)	24.6 ± 4.4	25.9 ± 6.0	0.3	n.s.
IE 90 (°)	28.0 ± 5.8	27.7 ± 9.1	0.1	n.s.
PS IE (°)	21.3 ± 4.2	23.0 ± 3.3	0.3	n.s.
PS ACC (mm/s^2^)	517.4 ± 200.9	615.2 ± 171.0	0.3	n.s.

Lateral bone bruise means bone bruise on femoral, tibial, or both compartments. None-to-deep bone bruise means ICRS grade 0–1–2, extensive-generalized bone bruise means ICRS grade 3–4. Data are presented as mean and standard deviation. n.s. means non-significant differences (*p* > 0.05)

**Table 4:** Intraoperative kinematics according to the presence of isolated lateral or medial + lateral bone bruise

	Extensive-generalized isolated lateral bone bruise (*n* = 9)	Extensive-generalized bicompartmental bone bruise (*n* = 7)	Rank-biserial correlation	*p* value
IE 30 (°)	21.6 ± 5.7	28.7 ± 3.6	0.6	0.012*
IE 90 (°)	22.4 ± 8.5	32.7 ± 3.7	0.7	0.003*
PS IE (°)	21.0 ± 3.2	23.6 ± 3.5	0.4	n.s.
PS ACC (mm/s^2^)	569.1 ± 151.7	613.6 ± 196.1	0.2	n.s.

Lateral or medial bone bruise means bone bruise on femoral, tibial, or both compartments. Isolated lateral bone bruise means ICRS grade 3–4 only on the lateral compartment. Bicompartmental bone bruise means ICRS grade 3–4 on both medial and lateral compartment. Data are presented as mean and standard deviation. Asterisks represent statistically significant differences (*p* < 0.05). n.s. means non-significant differences (*p* > 0.05)

## Discussion

The main finding of the present study was that severe bicompartmental BB was associated with higher intraoperative rotatory knee laxity (IE30, IE90), and that severity of bone edema on the medial tibial plateau had a direct correlation with intraoperative pivot shift in ACL-injured knee. No relationship was found between severe isolated lateral compartment edema and rotatory laxity (Figs. 2, 3). Moreover, high-grade edema of the lateral femoral condyle was associated with reduced preoperative clinical scores (KOOS-Symptoms).

The relationship between bone edema and rotatory knee laxity in acute ACL tear is a recently investigated topic. To our knowledge, only two studies with this interesting purpose were previously conducted [24, 30]. Song et al. [30] retrospectively investigated 193 ACL reconstructions: the patients were clinically evaluated under anesthesia before the surgery, and the presence and severity of BB were registered from their preoperative MRIs. The authors reported a correlation between severe edema of the lateral compartment (both tibia and femur) and high-grade pivot shift. They also found a positive correlation with an abnormal ALL signal. However, this last finding was affected by the low sensitivity of MRI for detecting ALL injury compared to other techniques, e.g., ultrasound imaging [4, 6, 8, 24]. Such evidence contrasts with a more recent study by Marot et al. [24]. The authors performed a prospective study on 61 patients with clinical examination under anesthesia and preoperative MRI and ultrasound imaging to inspect the BB. No positive correlation between lateral BB and ALL tear nor high-grade pivot shift was reported. However, a positive correlation between ALL tear and high-grade pivot shift was identified.

The latter studies performed a subjective analysis of rotatory instability, while the present study was the first one with precise quantification of intraoperative knee laxity. In line with Marot et al. [24], no correlation between intraoperative knee rotatory laxity and the severity of lateral contusion was observed.

The present study was also the first one investigating the involvement of medial compartment BB. Although the presence of BB on the postero-medial portion of the tibial plateau is less common than on the lateral femoral condyle or lateral tibial plateau, it could be observed in up to 60% of the patients' acute MRI [17]. Therefore, the involvement of the medial compartment should be considered when investigating the association between BB and rotatory knee instability. Interestingly, the present study highlighted a significant impact of medial compartment BB on intraoperative rotatory knee instability.

Recent studies investigated the etiopathogenesis of BB in the context of non-contact ACL injury [12, 16, 29]: femoral and tibial edemas’ surfaces were matched in MRI-based 3D models to reproduce contusion position. These studies reported a considerable amount of anterior tibial translation at the time the bones impact and a condition of pathological knee subluxation. It remains unclear whether this results from the dislocation or the relocation of the joint [12–14, 16, 29]. If occurring in knee relocation, a more severe joint subluxation could result in higher impact energy. This might cause high-grade edema and the medial compartment's involvement, which requires a greater tibial translation because of the geometry of the tibial plateau. The presence of bicompartmental BB could therefore be indicative of a more severe knee dislocation and secondary knee restrainers tears (e.g., the anterolateral complex).

The presence of BB on the medial compartment was also associated with a lateral femoral notch deeper than 2 mm. A recent paper demonstrated an association between a notch deeper than 2 mm and higher laxity during pivot shift in ACL-deficient knees with specificity between 94 and 96% [23]. The presence of both radiological signs could be useful in the preoperative selection of patients requiring an associated anterolateral procedure in ACL reconstruction. Future studies with greater sample size might clarify the role of simultaneous BB and deep lateral femoral notch in preoperative knee laxity.

Consideration should be done about meniscal RAMP lesions. The presence of postero-medial tibial plateau BB was proposed as an associated finding for these meniscal–capsular junction tears of the medial meniscus posterior horn, largely investigated in the last decade [2, 15, 19]. The relationship between RAMP and medial edema could be explained by hypothetical common pathogenesis, with high-energy knee subluxation in reduction damaging the meniscal–capsular junction before bone impact. In the present study, no RAMP tears were identified in the enrolled patients, making it impossible to relate medial BB and RAMP in the context of rotatory instability.

A lower KOOS score in patients with severe lateral femoral condyle edema was observed. The relationship between bone bruises and preoperative clinical scores has yet been investigated. A correlation between edema and patients’ clinical features was advocated; however, preoperative knee pain and function were usually related to soft tissue and cartilage lesions rather than bone contusion [7, 9, 27, 31]. Interestingly, our data showed a lower KOOS score in patients with severe lateral femoral condyle edema. Furthermore, because of the absence of correlation between meniscal lesions and BB, meniscal status could not have influenced such a finding.

The findings reported by the present study could be used by the orthopedic surgeons during the preoperative ACL reconstruction planning, to guide the graft choice, to decide if associated procedures are required and to set patients’ expectations.

However, the present study has several limitations. First, the sample size was smaller than that in the current literature on the same topic. This was due to the accurate methodology adopted: no previous studies approached the same issue by quantifying knee laxity with the gold standard device for intraoperative kinematics assessment. Second, the injury-to-MRI time (up to 3 months, 1.6 months on average) could be considered in the upper bounds for “acute” injury standards. However, previous studies investigating bone bruise prevalence and location in ACL injury included MRI evaluation performed up to 6 months from the time of injury [9]. Lastly, a subjective score (ICRS) was used to grade BB severity. However, such a score was used in previous studies, and the high inter-rater reliability limited the bias.

## Conclusion

A severe bicompartmental BB was related to higher rotatory instability in the intraoperative evaluation of ACL deficient knees. The severity of edema on the medial tibial plateau directly correlated with higher intraoperative pivot shift, and the size of edema on the lateral femoral condyle was associated with lower preoperative clinical scores.

## Supplementary Information

Below is the link to the electronic supplementary material.

Supplementary file1 (DOCX 14 kb)

## Abbreviations

ACL: Anterior cruciate ligament
ALL: Antero-lateral ligament
ICRS: International Cartilage Repair Society
IE30: Internal–external rotation at 30° of knee flexion
IE90: Internal–external rotation at 90° of knee flexion
PS IE: Internal–external rotation during pivot-shift test
PS ACC: Acceleration during pivot-shift test
