# Supplementary material for: Severe bicompartmental bone bruise is associated with rotatory instability in anterior cruciate ligament injury
Source: Knee Surg Sports Traumatol Arthrosc. 2021 Sep 7;30(5):1725–32. doi: 10.1007/s00167-021-06735-0 (PMC9033705; doi:10.1007/s00167-021-06735-0)
Supplement: Supplementary file 1 — Supplementary file1 (DOCX 14 kb) [file 167_2021_6735_MOESM1_ESM.docx]

| **Appendix A: Rank Correlations between bone bruise severity and kinematical or clinical outcomes** | | | | |
| --- | --- | --- | --- | --- |
|  | **Lateral Femoral Condyle** | **Medial Femoral Condyle** | **Lateral Tibial Plateau** | **Medial Tibial Plateau** |
| **Intraoperative kinematics** |  |  |  |  |
| IE 30 (°) | 0.13 | 0.01 | -0.02 | 0.35 |
| IE 90 (°) | -0.05 | -0.03 | -0.03 | 0.45* |
| PS IE (°) | 0.22 | 0.11 | 0.05 | 0.44* |
| PS ACC (mm/s^2^) | 0.24 | 0.16 | 0.21 | 0.39* |
| **Clinical outcomes (KOOS)** |  |  |  |  |
| PAIN | -0.14 | -0.10 | -0.05 | -0.04 |
| SYMPTOMS | -0.40* | -0.18 | -0.35 | -0.18 |
| ADL | -0.15 | -0.19 | -0.13 | 0.01 |
| SPORT | -0.22 | -0.28 | -0.17 | -0.15 |
| QoL | -0.01 | -0.07 | 0.10 | 0.05 |

Note: Asterisks represent statistically significant differences (p<0.05).
